# Profiling Commenters on Mental Health–Related Online Forums: A Methodological Example Focusing on Eating Disorder–Related Commenters

**DOI:** 10.2196/12555

**Published:** 2019-04-22

**Authors:** Duncan McCaig, Mark T Elliott, Cynthia SQ Siew, Lukasz Walasek, Caroline Meyer

**Affiliations:** 1 Warwick Manufacturing Group University of Warwick Coventry United Kingdom; 2 Department of Psychology University of Warwick Coventry United Kingdom; 3 Department of Psychology National University of Singapore Singapore Singapore; 4 Warwick Medical School University of Warwick Coventry United Kingdom; 5 Coventry and Warwickshire NHS Partnership Trust Coventry United Kingdom

**Keywords:** mental health, eating disorders, social media, social networks

## Abstract

**Background:**

Understanding the characteristics of commenters on mental health–related online forums is vital for the development of effective psychological interventions in these communities. The way in which commenters interact can enhance our understanding of their characteristics.

**Objective:**

Using eating disorder–related (EDR) forums as an example, this study detailed a methodology that aimed to determine subtypes of mental health–related forums and profile their commenters based on the other forums to which they contributed.

**Methods:**

The researchers identified all public EDR forums (with ≥500 contributing commenters between March 2017 and February 2018) on a large Web-based discussion platform (*Reddit*). A mixed-methods approach comprising network analysis with community detection, text mining, and manual review identified subtypes of EDR forums. For each subtype, another network analysis with community detection was conducted using the EDR forum commenter overlap between 50 forums on which the commenters also commented. The topics of forums in each detected community were then manually reviewed to identify the shared interests of each subtype of EDR forum commenters.

**Results:**

Six subtypes of EDR forums were identified, to which 14,024 commenters had contributed. The results focus on 2 subtypes—proeating disorder and thinspiration—and communities of commenters within both subtypes. Within the proeating disorder subtype, 3 communities of commenters were detected that related to the body and eating, mental health, and women, appearance, and mixed topics. With regard to the thinspiration group, 78.17% (849/1086) of commenters had also commented on pornographic forums and 16.66% (181/1086) had contributed to proeating disorder forums.

**Conclusions:**

The article exemplifies a methodology that provides insight into subtypes of mental health–related forums and the characteristics of their commenters. The findings have implications for future research and Web-based psychological interventions. With the publicly available data and code provided, researchers can easily reproduce the analyses or utilize the methodology to investigate other mental health–related forums.

## Introduction

### Background

Compared with clinician-delivered psychological interventions, Web-based interventions targeting mental health conditions offer several benefits, such as cost-effectiveness [1], and potentially reach a larger number of the target population [2]. However, for these interventions to be effective, it is important that users adhere to them. A recent review indicated that several characteristics (eg, gender and impersonality of intervention content) are important predictors of adherence to Web-based psychological interventions [3]. Therefore, if adherence to Web-based psychological interventions is to be improved, a clearer understanding of the characteristics of target populations (eg, online mental health–related communities) is vital. This study has used the example of eating disorder–related (EDR) forums to outline a reproducible methodology that can enhance our understanding of the characteristics of online mental health–related communities.

### Eating Disorder–Related Forums

EDR forums are easily accessible online, and their content can be defined broadly as either *proeating disorder* or *prorecovery* [4]. Proeating disorder content encourages the enactment of eating-disordered behaviors (eg, fasting and excessive exercise) without indicating a desire for recovery and typically portrays eating disorders as a lifestyle choice rather than a mental health condition [4]. The term *thinspiration* is often used to refer to proeating disorder material (eg, photos) that encourages eating disordered behaviors [5]. In contrast, prorecovery (or *antieating disorder*) content encourages recovery from eating disorders or confronting eating-disordered behaviors [4]. In addition to the proeating disorder or prorecovery distinction, EDR forums can also be characterized as relating to eating disorders in general or specific diagnostic categories (eg, anorexia nervosa, bulimia nervosa, or binge eating disorder).

A deeper understanding of proeating disorder forums is particularly important [6]. Emphasizing this importance, semistructured interviews with current eating disorder patients found that they perceived proeating disorder websites as having reinforced and maintained their eating disorders [7]. Furthermore, a recent meta-analysis [8] showed that greater engagement with proeating disorder forums is associated with increased body dissatisfaction, dieting, and negative affect but not bulimic symptoms. Despite this issue’s importance, few studies have attempted to elucidate the characteristics of people who engage with EDR forums.

#### Survey-Based Research

Most existing studies attempting to characterize users of EDR forums have used survey-based methods and samples recruited directly from EDR forums or student samples. For example, Peebles et al [9] recruited participants from proeating disorder websites and found that they were predominantly young women within the healthy body mass index range. Over 70% of the respondents also indicated that they had purged, binged, or used laxatives to control their weight. Exemplifying the use of student samples, Harper et al [10] asked undergraduate women to complete self-report questions about viewing EDR websites. Compared with controls who had not viewed EDR websites, participants who had viewed proeating disorder websites reported higher appearance dissatisfaction, dietary restriction and bulimic symptoms, and more frequent viewing of cosmetic surgery websites.

Although the research detailed above provides insight into commenters’ characteristics using standardized self-report scales, the studies’ samples are unlikely representative of everyone who engages with EDR forums. For example, survey respondents on forums might differ from those who do not respond [9] and relatively small student populations might not be representative of the actual forum users [10].

#### Textual Analyses of Online Content

Textual analyses of online content offer an alternative methodology to surveys and complement findings obtained through self-report measures. Such analyses can be approached in different ways, including manual qualitative methods (eg, thematic analysis and content analysis) and computerized methods (eg, word counts and topic modelling). However, these approaches all address a similar aim in identifying themes discussed on EDR forums.

Through using manual qualitative methods, researchers have found that most EDR forum commenters are women [4] and that eating and shape concerns are the most commonly expressed symptoms in a proeating disorder forum [11]. Despite variability between studies, applications of computerized word count methods have indicated that prorecovery and proeating disorder commenters differ in terms of factors such as affect and self-directed attention [12,13] and mentions of fitness tracking technology [14]. A combination of both manual and computerized textual analyses has also indicated that recovery is less frequently mentioned by commenters in the least recovery-focused eating disorder stages of change (ie, precontemplation and relapse) [15]. Recently, Moessner et al [16] employed sophisticated computerized topic modelling to characterize a proeating disorder community in terms of the themes that its commenters discuss, including feedback and social support and weight gain or loss.

#### Interaction Between Forum Users

A complementary approach to textual analyses is to explore the way in which users on a forum interact. In the same study detailed above, Moessner et al [16] investigated how commenters within a proeating disorder forum interacted and were able to identify particularly influential commenters. In contrast, 2 studies have explored the interaction between communities of commenters on *Twitter* [13,17]. Tiggemann et al [17] theoretically selected and compared thinspiration and *fitspiration* (ie, content encouraging health and fitness) posts on *Twitter*. During a 2-week period, the researchers identified users that included thinspiration or fitspiration tags in their posts and found minimal overlap (ie, interaction) between commenters in the 2 communities. Using a more data-driven approach, Wang et al [13] detected 2 communities of commenters who posted comments with EDR tags. The 2 communities were found to reflect proeating disorder and prorecovery stances, with network analyses indicating minimal overlap between the communities.

Although Moessner et al [16] explored the user interaction *within* a forum, Tiggemann et al [17] and Wang et al [13] investigated the user interaction *between* 2 communities. However, these approaches could be extended to explore the overlap between, theoretically, an infinite number of communities. Although researchers have previously used network analyses to investigate traffic between over 500 EDR websites [18], this has not been done with online discussion forums nor at the level of the individual user.

### Reddit

In February 2018, *Reddit* was the sixth most visited website in the world, with 234 million unique visitors [19]. *Reddit* is a large Web-based discussion platform comprising thousands of forums (ie, *subreddits*) and is perfectly suited to investigate the overlap between online communities on a large scale. Each subreddit relates to a specific topic (eg, politics or films) and can be conceptualized as a community of people with a shared interest. A member of a subreddit can start a conversation (ie, *thread*) or join a conversation by responding to an existing comment. *Reddit* can be viewed as a microcosm of the internet, as users are likely to engage only with a relatively small number of subreddits that interest them. *Reddit* therefore provides a unique opportunity to explore how online forums are related in terms of whether the same people comment on them. Furthermore, by identifying a group of commenters and identifying the other forums to which they contribute, the communities to include in analyses can be determined in a largely data-driven way, in contrast to the theoretical approach used in previous studies [17,18]. Finally, as eating disorders are the explicit focus of several subreddits and have been the focus of previous studies [11,12,16], *Reddit* presents a particularly beneficial opportunity to investigate the characteristics of EDR forums in greater detail.

### Objectives

Although it is common for studies to consider EDR online discussion forums in isolation [11,14,16], recent studies have investigated how similar forums overlap in terms of commenters [17]. This study built on the latter approach and used publicly available data from *Reddit* [20] to achieve 2 separate objectives, as detailed below.

#### Objective 1: Determining Subtypes of Eating Disorder–Related Subreddits

The first objective of this study was to identify subtypes of EDR forums on *Reddit*. To achieve this, large EDR subreddits were identified, and the way in which they overlapped with regard to commenters was calculated. A mixed-methods approach was then applied, comprising network analysis with community detection, text mining, and manual review of the EDR subreddits’ focuses. This enabled the grouping of EDR subreddits into subtypes and, as a result, facilitated the elucidation of thematic heterogeneity in EDR subreddits (eg, proeating disorder and prorecovery). The output regarding Objective 1 also determined the grouping of subreddits for the analyses relating to Objective 2.

#### Objective 2: Profiling Eating Disorder–Related Commenters Based on Contributions to Ancillary Subreddits

The second objective of this study was to profile each subtype of EDR forums (see Objective 1) in terms of their main interests, as represented by the topics of subreddits to which the commenters also contributed. To achieve Objective 2, all the other (public) subreddits to which EDR commenters had contributed (*ancillary subreddits*) were identified. A mixed-methods approach was then conducted consisting of network analysis with community detection and manual review of the ancillary subreddits’ topics. The overall topics of the subreddits comprising each detected community then enabled the profiling of groups of EDR commenters in terms of the other topics in which they were interested.

## Methods

### Corpus Selection and Data Analysis

All public *Reddit* comments, excluding the initial post to which commenters respond, are regularly archived and freely available [20]. Although the archive includes all comments from December 2005, this study’s corpus comprises all comments posted between March 2017 and February 2018 (inclusive). This represents the 1-year period preceding the most recent month’s data that were available when beginning the study. As publicly available data were used, this study was outside the remit of the University of Warwick’s Biomedical and Scientific Research Ethics Committee, from whom an exemption from ethical review was obtained. All data were extracted, preprocessed, and analyzed with the Python (Python Software Foundation) programming language [21], except where otherwise stated. The code used in this study is available as Multimedia Appendix 1 and can be used to replicate the analyses.

### Objective 1: Determining Subtypes of Eating Disorder–Related Subreddits

A list of search terms was created to identify EDR subreddits, which is provided in Multimedia Appendix 2. This list was generated through a consultation of EDR sections of 2 clinical references, DSM-V and ICD-10 [22,23], and previous research concerning EDR online communities [14,24]. Search terms were developed that related to (1) eating disorders in general (eg, eating disorder); (2) specific eating disorder diagnostic categories (eg, anorexia nervosa, bulimia nervosa, or binge eating disorder); or (3) online content associated with eating disorders (eg, thinspiration). Subreddits were included that contained at least 1 search term in their name or brief description but excluded if they (1) were unrelated to eating disorders (eg, *Anorexiclizardpeople*) or (2) were private. For each subreddit identified through this search and inclusion strategy, each comment and commenter’s name were then extracted from the *Reddit* data [20]. To focus the analyses on the largest EDR subreddits, any subreddits with fewer than 500 commenters contributing within the 1-year period were excluded. The *Reddit* commenters *AutoModerator* and *[deleted]* were not included in this count and were excluded from all subsequent analyses.

A mixed-methods approach was then used to identify subtypes of the EDR subreddits. This approach comprised 3 techniques that are described below and were conducted in the order presented: (1) network analyses with community detection; (2) text mining; and (3) manual review of EDR subreddits’ focuses.

#### Network Analyses With Community Detection

To conduct the network analyses with community detection, a list of commenters was compiled separately for each of the included EDR subreddits. For each pairwise comparison of the subreddits (eg, *subreddit A* compared with *subreddit B*), the proportion of each subreddit’s commenters who had posted on the other subreddit was calculated, with the result ranging from 0 (no commenters overlap) to 1 (all commenters overlap). For example, a proportion of 0.4 (40/100) of *subreddit A* ’s commenters might post on *subreddit B*, whereas 0.8 (40/50) of *subreddit B* ’s commenters post on *subreddit A*. The mean of these 2 proportions (ie, 0.6) was calculated to account for the differences in the number of commenters on each subreddit. A matrix was then created using all these pairwise comparisons, where each cell within the matrix represented the mean commenter overlap between each pairing of the EDR subreddits.

Using this matrix, a weighted and undirected (ie, associative) network analysis was conducted using the *qgraph* package [25] for *R* statistical software [26]. Many techniques exist for the purpose of detecting communities (ie, subreddits with similar commenters) within networks. The *walktrap* algorithm [27] was used for this purpose, as it is recommended for networks with fewer than 1000 nodes (ie, subreddits) [28] and was observed to detect communities more reliably than other suitable algorithms [28].

As with individual subreddits, the detected communities might also overlap in terms of commenters (ie, a commenter might contribute to subreddits from more than one community). As such, for each detected community, commenter lists were compiled and used to calculate the mean commenter overlap between communities in the same way as detailed above for the pairwise comparisons of subreddits. In the event of more than 2 communities being detected, the *VennDiagram* *R* package [29] was used to visualize these commenter overlaps using unscaled Venn diagrams.

#### Text Mining

An existing text-mining approach was used to establish the degree to which each EDR subreddit was recovery-focused [14]. For each EDR subreddit, the percentage of its comment threads that contained at least 1 recovery term (ie, *recovery*, *recover*, *recovers*, *recovered*, and *recovering*) was calculated. A higher percentage of threads containing at least 1 reference to recovery was interpreted as representing a greater recovery focus, in line with findings that recovery is less frequently mentioned by people in the precontemplation or relapse eating disorder stages of change [15]. For example, between May 2015 and January 2018 (inclusive), 10% of the subreddit *proED*’s threads contained a reference to recovery, compared with 50% of the subreddit *EatingDisorders’* threads [14]. As this represented a 40% difference between the two, *EatingDisorders* was interpreted as having a greater recovery focus than *proED.*

#### Manual Review of Eating Disorder–Related Subreddits’ Focuses

A manual review of each EDR subreddit’s name and brief description was undertaken to define whether each EDR subreddit is related to (1) eating disorders in general; (2) specific eating disorder diagnostic categories (eg, anorexia nervosa, bulimia nervosa, or binge eating disorder); or (3) online content associated with eating disorders (eg, thinspiration). This step reflects the previously detailed distinctions used in the generation of the initial search terms. The definitions were then considered together with the results from the previous 2 steps (ie, network analysis with community detection and text mining) to guide the categorization of the EDR subreddits. For example, 2 EDR subreddits specific to anorexia nervosa would have been categorized differently if they were present in 2 distinct communities representing different levels of recovery focus. All of the researchers reached an agreement on the categorization of EDR subreddits at this stage.

### Objective 2: Profiling Eating Disorder–Related Commenters Based on Contributions to Ancillary Subreddits

For each of the subtypes of EDR subreddits identified through the previously described methods (Objective 1), a list was compiled of all the commenters who had contributed within the 1-year period to at least 1 subreddit within the subtype (excluding *AutoModerator* and *[deleted]* commenters). To focus this report on the largest subtypes of EDR subreddits, only subtypes with 1000 or more commenters were included in the analyses. The following analyses were repeated separately for each included subtype of EDR subreddits.

Using the respective list of commenters for the EDR subtype, all of the other subreddits to which the commenters had contributed (ie, ancillary subreddits) in the 1-year period were identified. Any ancillary subreddits to which fewer than 1% of the EDR subtype’s commenters had contributed were excluded at this stage. This exclusion was made as ancillary subreddits with so few EDR commenters would not have been included in the final steps of the analysis (detailed below) and therefore represented unnecessary data to extract.

The ancillary subreddits were then ranked separately in the descending order of (1) the number of the EDR subtype’s commenters who had contributed to each ancillary subreddit and (2) the proportion of each ancillary subreddit’s total commenters (ie, not only the EDR subtype’s commenters) that had also commented on at least 1 subreddit within the EDR subtype. For each ancillary subreddit, the mean of these 2 ranks was then calculated. Owing to the large number (ie, tens of thousands) of ancillary subreddits associated with each EDR subtype and to improve the interpretability of the results, the mean rank was used to identify the most representative ancillary subreddits for inclusion in the following analyses. Specifically, the 50 ancillary subreddits with the highest mean rank were included, resulting in the inclusion of ancillary subreddits that were both large in size (ie, comprised many commenters) and included a large proportion of the EDR subtype’s commenters. This avoided the inclusion of ancillary subreddits that were very large in terms of the number of commenters but of which the EDR subtype’s commenters comprised a very small proportion (eg, general subreddits, such as *AskReddit*). At the same time, this step also avoided the inclusion of very small subreddits that had very high proportions of the EDR subtype’s commenters (eg, subreddits comprising a few commenters who had all contributed to at least 1 subreddit within the EDR subtype). As this threshold (ie, the top 50 subreddits) was used solely to facilitate a clear interpretation of the results, extensions of this study could set different thresholds to explore the communities at varying levels of detail.

A mixed-methods approach was then used to profile the EDR subtype’s commenters in terms of their main interests, as represented by the thematic focuses of the ancillary subreddits. This approach comprised 2 techniques that are described below and were conducted in the order presented: (1) network analyses with community detection and (2) a manual review of the ancillary subreddits’ topics.

#### Network Analyses With Community Detection

The network and community detection analyses were conducted in the same way as detailed for Objective 1. The only difference was that the commenter overlaps (relating to both the ancillary subreddits and detected communities) were calculated using only the EDR subtype’s commenters (ie, excluding commenters on each ancillary subreddit who had not contributed to at least 1 of the EDR subtype’s subreddits).

#### Manual Review of Ancillary Subreddits’ Topics

A manual review of each ancillary subreddit’s name and brief description was undertaken to describe each subreddit’s general topic. For example, the general topic of the subreddit *loseit* was described as *weight loss*. All ancillary subreddits comprising the detected communities were then reviewed, and a label was produced to represent the general content of each community. For example, a community containing ancillary subreddits that related to eating behaviors and weight loss was labelled *Eating/Body*. To ensure transparency at every stage of this process, the name and labels of all included ancillary subreddits are presented in tables in the Results section, along with a summary of how each label for the detected communities was generated.

## Results

### Objective 1: Determining Subtypes of Eating Disorder–Related Subreddits

**Figure 1:**
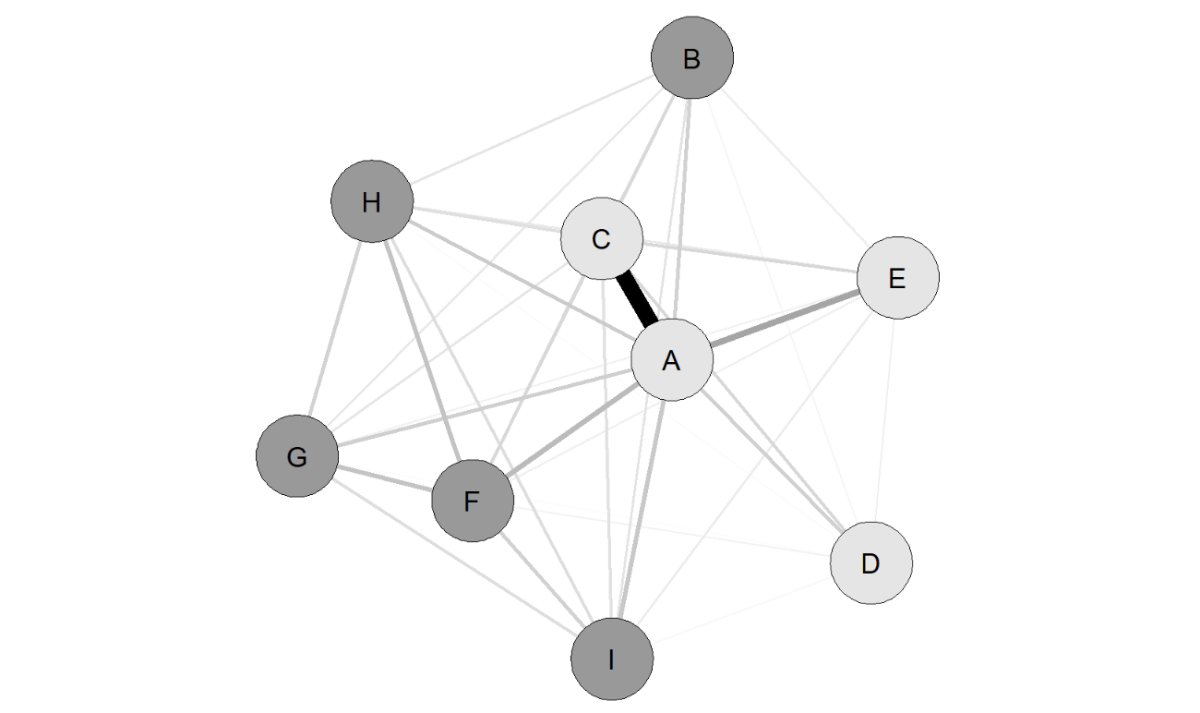
Eating disorder–related subreddits network. Letters correspond to eating disorder–related subreddits (A=*proED*, B=*BingeEatingDisorder*, C=*ProEDmemes*, D=*thinspo*, E=*MyProAna*, F=*fuckeatingdisorders*, G=*EatingDisorders*, H=*eating_disorders*, and I=*bulimia*). Light-gray circles represent community 1 (low recovery focus), and dark-gray circles represent community 2 (high recovery focus). Thickness of the lines represents the mean degree of commenter overlap between each pair of subreddits (thicker line=larger overlap).

The search and inclusion strategy led to the identification of 50 EDR subreddits, a list of which is provided in Multimedia Appendix 2. Following the exclusion of any subreddits with fewer than 500 commenters, 9 EDR subreddits were identified: *BingeEatingDisorder*, *bulimia*, *EatingDisorders*, *eating_disorders*, *fuckeatingdisorders*, *MyProAna*, *proED*, *ProEDmeme* s, and *thinspo*. In total, 14,024 commenters posted on these 9 EDR subreddits. Of these commenters, 0.69% (97/14,024) included the term *bot* within their account name, with these commenters contributing a mean of 9 comments (SD 16; median 3, minimum 1, maximum 94) to the EDR subreddits. The network analysis with community detection corresponding to the 9 EDR subreddits is presented in Figure 1.

Figure 1 shows that the community detection algorithm identified 2 communities in the EDR subreddits network. The text-mining analyses found that community 1 (light-gray circles) comprised the 4 EDR subreddits with the lowest percentage of threads mentioning recovery: *MyProAna* (9.9%; 47/471), *proED* (11.72%; 2390/20,396), *ProEDmemes* (2.38%; 39/1639), and *thinspo* (0.23%; 3/1323). In contrast, community 2 (dark-gray circles) comprised the 5 EDR subreddits with the highest percentage of threads mentioning recovery: *BingeEatingDisorder* (19.30%; 411/2129), *bulimia* (40.0%; 167/417), *eating_disorders* (32.1%; 179/557), *EatingDisorders* (46.3%; 192/414), and *fuckeatingdisorders* (45.5%; 191/419). These findings supported a conceptualization of community 1 comprising *low recovery-focus* EDR subreddits and community 2 comprising *high recovery-focus* EDR subreddits.

Of the 14,024 commenters, 65.97% (9252/14,024) *only* posted on subreddits within the low recovery-focus community, whereas 28.69% (4023/14,024) *only* commented on subreddits within the high recovery-focus community. However, 5.34% commenters (749/14,024) posted on subreddits within *both* communities, indicating relatively little commenter overlap between the communities.

In addition to the degree of recovery focus, the EDR subreddits also differed in terms of whether they concerned eating disorders in general (eg, *EatingDisorders*), a specific eating disorder diagnostic category (eg, *BingeEatingDisorder*), or online content associated with eating disorders (eg, *thinspo*). Each subreddit’s focus was therefore used to categorize the subreddits within each detected community. Consequently, the low recovery-focus community comprised 3 subtypes of EDR subreddits: *pro-eating disorder*, consisting of *proED* and *ProEDmemes* (8166 commenters); *thinspiration*, consisting of *thinspo* (1580 commenters); and *pro-anorexia nervosa*, consisting of *MyProAna* (731 commenters). As with the low recovery-focus community, the high recovery-focus community comprised 3 subtypes: *pro-recovery eating disorder*, consisting of *EatingDisorders*, *eating_disorders,* and *fuckeatingdisorders* (1986 commenters); *pro-recovery binge eating disorder*, consisting of *BingeEatingDisorder* (2520 commenters); and *pro-recovery bulimia nervosa*, consisting of *bulimia* (524 commenters).

### Objective 2: Profiling Eating Disorder–Related Commenters Based on Contributions to Ancillary Subreddits

To focus this report on the largest subtypes of EDR subreddits, subtypes with fewer than 1000 commenters were excluded at this stage (ie, *MyProAna* and *bulimia*). Consequently, the analyses regarding Objective 2 were conducted for 4 subtypes of EDR subreddits: 2 that are conceptualized as low recovery focus (ie, proeating disorder subreddits and thinspiration) and 2 that are conceptualized as high recovery focus (ie, prorecovery eating disorder subreddits and prorecovery binge eating disorder). To examine the networks in sufficient detail and owing to the particular importance of the proeating disorder communities [6], only the analyses for the low recovery-focus subtypes are presented below. The analyses for the high recovery-focus subtypes are provided in Multimedia Appendix 2 (supplementary analyses).

#### Low Recovery Focus: Proeating Disorder

**Figure 2:**
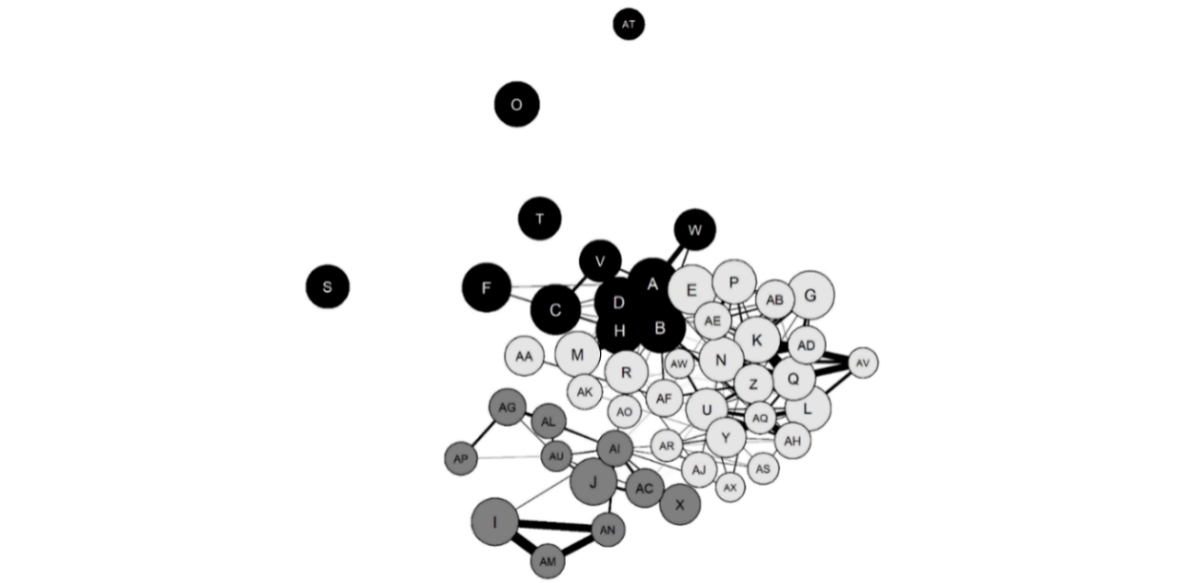
Proeating disorder network. Letters correspond to ancillary subreddits. Black circles represent community 1 (*Eating/Body*), dark-gray circles represent community 2 (*Mental health*), and light-gray circles represent community 3 (*Women/Appearance/Mixed*). The size of circles represents the ancillary subreddit mean rank (larger circle=higher rank), and thickness of the lines represents the mean degree of commenter overlap between each pair of subreddits (thicker line=larger overlap). No lines representing <0.25 mean commenter overlap are displayed.

In total, 974 ancillary subreddits had been contributed to by at least 1% (82) of the 8166 commenters associated with the *pro-eating disorder* subtype (ie, *proED* and *ProEDmemes*). 50 ancillary subreddits were identified, on which 61.95% (5059/8166) of the proeating disorder subtype’s commenters also posted. The network analysis with community detection is presented in Figure 2, with a summary of the 50 ancillary subreddits presented in Table 1.

**Table 1:** Names and descriptions of ancillary subreddits on which proeating disorder commenters posted.

Community	Rank	Label	Subreddit name	Description
1^a^	1	A	1200isplenty	1200kcal daily energy intake
1	2	B	fatlogic	Weight-loss
1	3	C	fasting	Fasting
1	4	D	progresspics	Photos of “body transformations”
1	6	F	BingeEatingDisorder	Binge eating disorder (19.08% threads mention recovery)
1	8	H	loseit	Weight-loss
1	15	O	EDFood	Food in the context of eating disorders
1	19	S	MyProAna	Anorexia nervosa (10.26% threads mention recovery)
1	20	T	thinspo	Thinspiration (0.24% threads mention recovery)
1	22	V	intermittentfasting	Fasting
1	23	W	1200isjerky	1200kcal daily energy intake
1	46	AT	proEDadults	Eating Disorders
2^b^	9	I	selfharm	Self-harm
2	10	J	BPD	Borderline personality disorder
2	24	X	bipolar	Bipolar disorder
2	29	AC	morbidquestions	Ask “dark questions”
2	33	AG	depression	Depression
2	35	AI	SanctionedSuicide	Suicide
2	38	AL	Anxiety	Anxiety
2	39	AM	selfharmpics	Self-harm
2	40	AN	MadeOfStyrofoam	Self-harm
2	42	AP	SuicideWatch	Suicide
2	47	AU	mentalhealth	Mental health
3^c^	5	E	xxfitness	Female fitness
3	7	G	femalefashionadvice	Advice on female fashion
3	11	K	MakeupAddiction	Make-up addiction
3	12	L	muacirclejerk	Make-up addiction
3	13	M	fatpeoplestories	Stories about “fat people”
3	14	N	SkincareAddiction	“Everything skincare”
3	16	P	TheGirlSurvivalGuide	“A survival guide of ‘life pro-tips’ for the everyday girl”
3	17	Q	muacjdiscussion	Make-up addiction
3	18	R	vegan	Veganism
3	21	U	badwomensanatomy	Women’s anatomy
3	25	Y	awfuleyebrows	Photos of eyebrows that are judged to be “embarrassing, ugly, and downright weird”
3	26	Z	TrollXChromosomes	Women
3	27	AA	raisedbynarcissists	“Support group for people raised by (or being raised by) a narcissistic parent”
3	28	AB	FancyFollicles	Hair
3	30	AD	AsianBeauty	Beauty brands, cosmetics and skincare from Asia
3	31	AE	AskWomen	Ask women about any subject
3	32	AF	childfree	People who do not have or want children
3	34	AH	antiMLM	Multi-level marketing schemes
3	36	AJ	piercing	Piercing
3	37	AK	amiugly	Commenter posts photo and asks for feedback on appearance
3	41	AO	vegetarian	Vegetarianism
3	43	AQ	Youniqueamua	Make-up addiction
3	44	AR	Shoplifting	Shoplifting
3	45	AS	thesims	Computer game (life simulation game)
3	48	AV	BeautyGuruChatter	Discuss beauty “influencers” and “YouTubers”
3	49	AW	bulletjournal	Method of organisation
3	50	AX	actuallesbians	Cis- or trans-lesbians

^a^Community 1=*Eating/Body.*

^b^Community 2=*Mental health.*

^c^Community 3=*Women/Appearance/Mixed*.

As shown in Figure 2 and Table 1, the algorithm detected 3 communities within the proeating disorder network. Community 1 (black circles) was labelled *Eating/Body* as the ancillary subreddits were related to restrictive eating (eg, *1200isplenty*, *fasting*, and *intermittentfasting*), weight loss or body transformations (eg, *loseit*, *fatlogic*, and *progresspics*), or eating disorders (ie, *BingeEatingDisorder*, *MyProAna*, and *proEDadults*). Community 2 (dark gray circles) was labelled *Mental health* as the subreddits mainly related to mental health conditions (eg, *depression*, *Anxiety*, and *bipolar*) or related issues (eg, *selfharm*, *SanctionedSuicide*, and *SuicideWatch*). Community 3 (light-gray circles) was labelled *Women/Appearance/Mixed* as the subreddits related to women (eg, *xxfitness*, *TheGirlSurvivalGuide*, and *AskWomen*), appearance (eg, *MakeupAddiction*, *BeautyGuruChatter*, and *amiugly*), or mixed topics (eg, *vegan*, *childfree*, and *raisedbynarcissists*).

**Figure 3:**
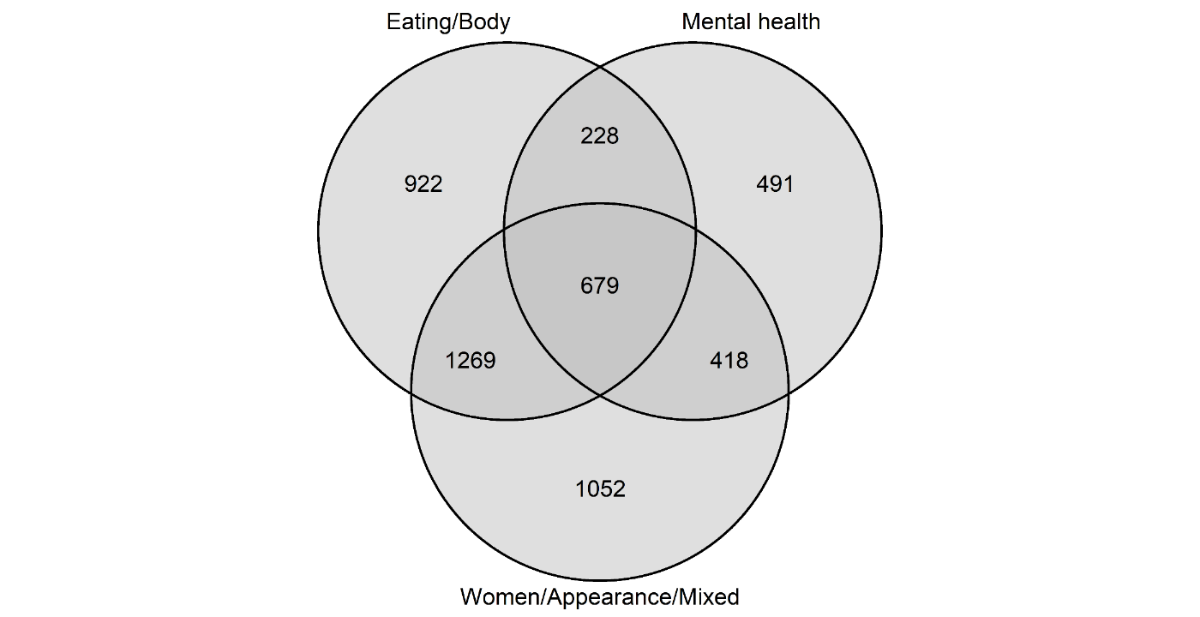
Commenter overlap between proeating disorder communities. Values represent the number of commenters in the proeating disorder network (n=5059) who posted in the 3 communities (represented by the 3 circles). Values in overlapping areas indicate the number of commenters who posted in 2 or more communities. The areas of circles are unscaled and do not represent the size of communities.

Of the 5059 proeating disorder commenters, 67.56% (3418) also posted on ancillary subreddits within the Women/Appearance/Mixed community, compared with 61.24% (3098) and 35.90% (1816) in the Eating/Body and Mental health communities, respectively. Figure 3 presents the commenter overlaps between the 3 proeating disorder communities.

#### Low Recovery Focus: Thinspiration

**Figure 4:**
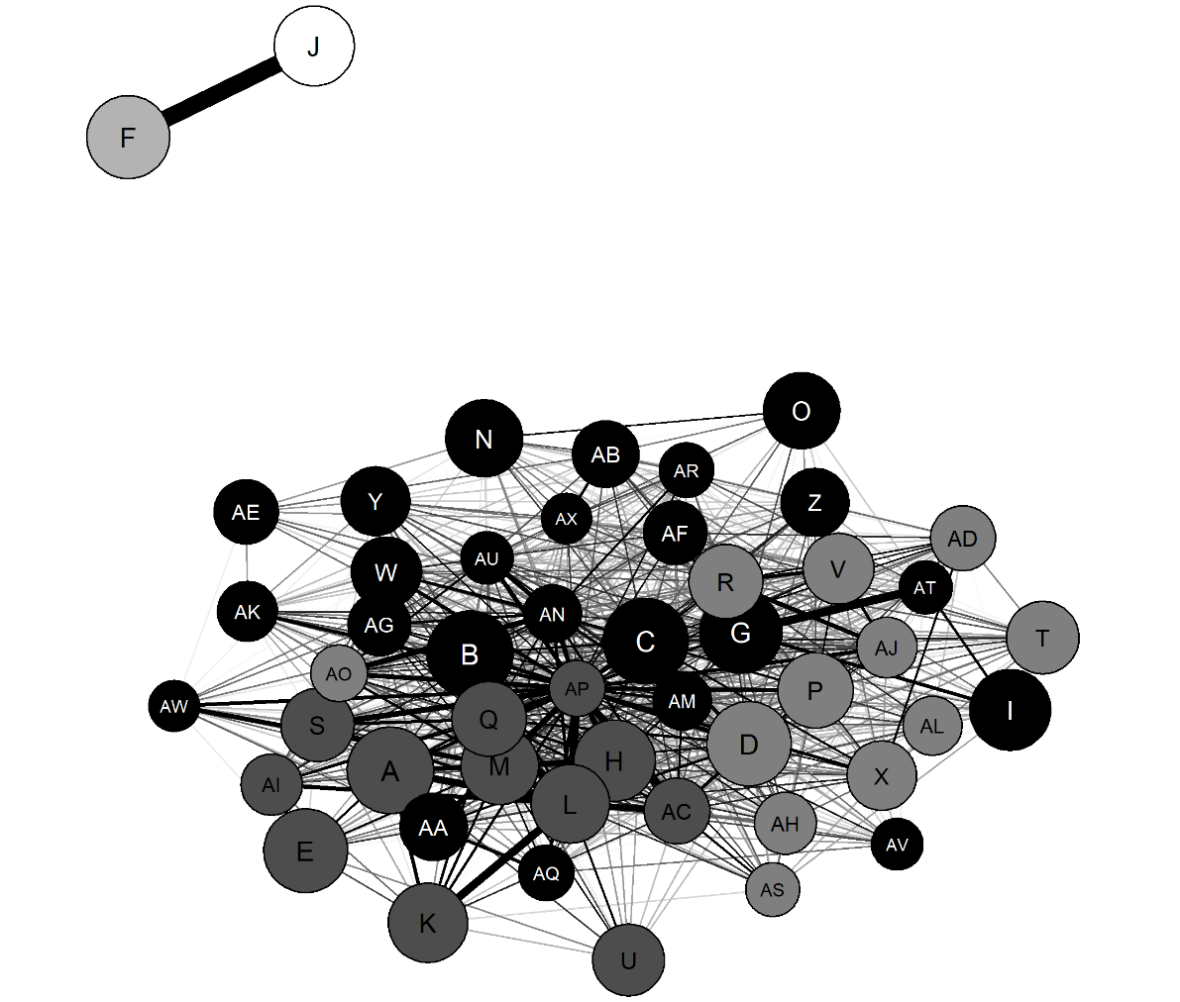
Thinspiration network. Letters correspond to ancillary subreddits. Black circles represent community 1 (*Pornography: 1*), dark-gray circles represent community 2 (*Pornography: young/small*), mid-gray circles represent community 3 (*Pornography: 2*), the light-gray circle represents community 4 (*ProEDmemes*), and the white circle represents community 5 (*proED*). The size of circles represents the ancillary subreddit mean rank (larger circle=higher rank), and thickness of the lines represents the mean degree of commenter overlap between each pair of subreddits (thicker line=larger overlap). No lines representing <0.25 mean commenter overlap are displayed.

In total, 3932 ancillary subreddits had been contributed to by at least 1.01% (16) of the 1580 commenters associated with the *thinspiration* subtype (ie, *thinspo*). A total of 50 ancillary subreddits were identified on which 68.73% (1086/1580) thinspiration subtype’s commenters also posted. The network analysis with community detection is presented in Figure 4, with a summary of the 50 ancillary subreddits presented in Table 2.

As shown in Figure 4 and Table 2, the algorithm detected 5 communities within the thinspiration network. Community 1 (black circles) was labelled *Pornography: 1* as the ancillary subreddits were all pornographic in nature. Community 2 (dark-gray circles) was labelled *Pornography: young/small* as it mainly comprised pornographic subreddits that explicitly referred to women being young (eg, *LegalTeens* and *18_19*) or small (eg, *xsmallgirls*, *TinyTits,* and *dirtysmall*). Community 3 (mid-gray circles) was labelled *Pornography: 2* as all the subreddits were pornographic. Community 4 (light-gray circle) was labelled *ProEDmemes* as it comprised only 1 subreddit, *ProEDmemes*. Similarly, community 5 (white circle) was labelled *proED*, as it consisted of *proED* only.

**Table 2:** Names and descriptions of ancillary subreddits on which thinspiration commenters posted.

Community	Rank	Label	Subreddit name	Description
1^a^	2	B	CuteLittleButts	Pornography^b^
1	3	C	SexyTummies	Pornography
1	7	G	fitgirls	Pornography
1	9	I	SkinnyWithAbs	Pornography
1	14	N	(omitted from report)^c^	Pornography
1	15	O	(omitted from report)^c^	Pornography
1	23	W	Ifyouhadtopickone	Pornography
1	25	Y	WtSSTaDaMiT	Pornography
1	26	Z	theratio	Pornography
1	27	AA	HugeDickTinyChick	Pornography
1	28	AB	goddesses	Pornography
1	31	AE	NSFWfashion	Pornography
1	32	AF	lingerie	Pornography
1	33	AG	FestivalSluts	Pornography
1	37	AK	uncommonposes	Pornography
1	39	AM	ginger	Pornography
1	40	AN	HappyEmbarrassedGirls	Pornography
1	43	AQ	distension	Pornography
1	44	AR	Ohlympics	Pornography
1	46	AT	hardbodies	Pornography
1	47	AU	girlskissing	Pornography
1	48	AV	GirlswithNeonHair	Pornography
1	49	AW	whenitgoesin	Pornography
1	50	AX	PrettyGirls	Pornography
2^d^	1	A	xsmallgirls	Pornography
2	5	E	skinnytail	Pornography
2	8	H	funsized	Pornography
2	11	K	aa_cups	Pornography
2	12	L	TinyTits	Pornography
2	13	M	dirtysmall	Pornography
2	17	Q	LegalTeens	Pornography
2	19	S	18_19	Pornography
2	21	U	petite	Pornography
2	29	AC	palegirls	Pornography
2	35	AI	tanlines	Pornography
2	42	AP	adorableporn	Pornography
3^e^	4	D	datgap	Pornography
3	16	P	bodyperfection	Pornography
3	18	R	tightdresses	Pornography
3	20	T	legs	Pornography
3	22	V	randomsexiness	Pornography
3	24	X	BonerMaterial	Pornography
3	30	AD	bikinis	Pornography
3	34	AH	nsfwoutfits	Pornography
3	36	AJ	girlsinyogapants	Pornography
3	38	AL	pokies	Pornography
3	41	AO	StraightGirlsPlaying	Pornography
3	45	AS	SexyFrex	Pornography
4^f^	6	F	ProEDmemes	Eating disorders (2.42% threads mention recovery)
5^g^	10	J	proED	Eating disorders (11.75% threads mention recovery)

^a^Community 1=*Pornography: 1.*

^b^The term *Pornography* is used generally to describe any subreddit featuring material for the ostensibly exclusive purpose of sexual arousal.

^c^The name of the subreddit relates to a specific person and is omitted from the report.

^d^Community 2=*Pornography: young/small.*

^e^Community 3=*Pornography: 2.*

^f^Community 4=*ProEDmemes*.

^g^Community 5=*proED*.

**Figure 5:**
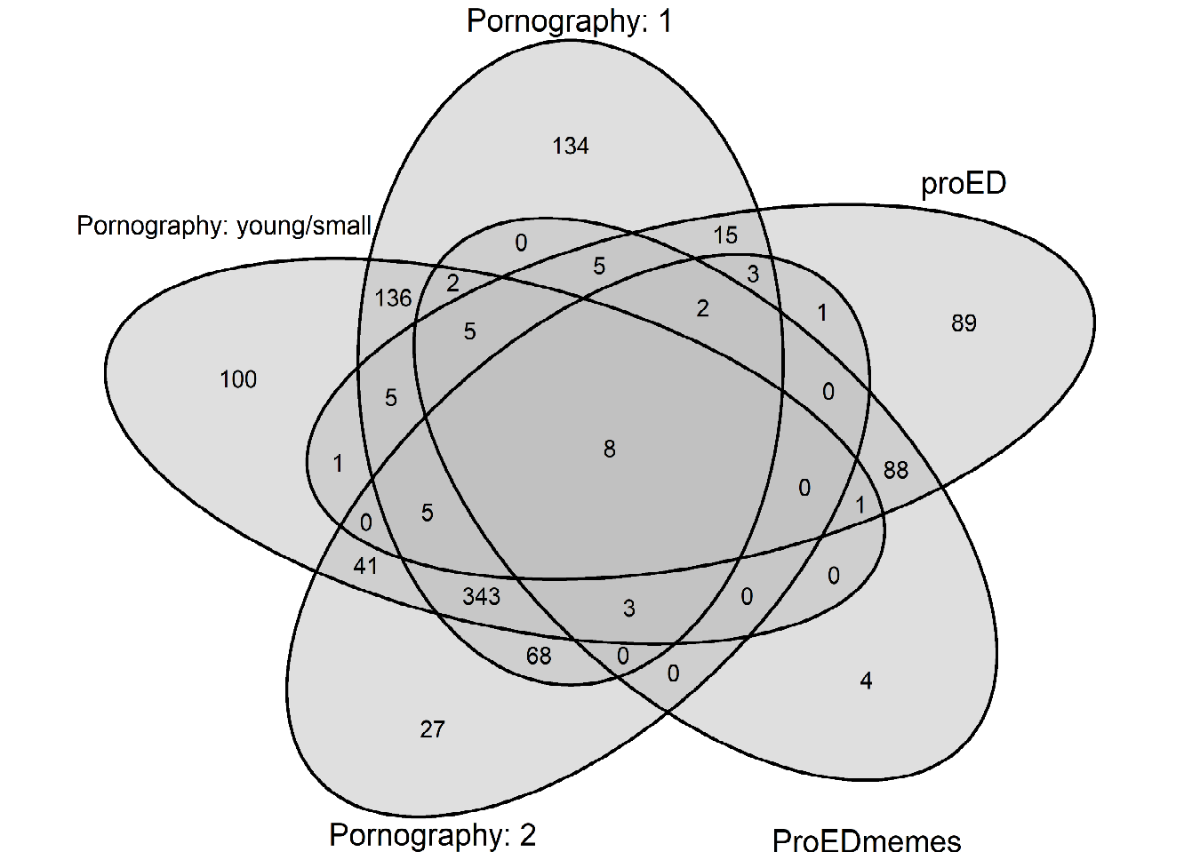
Commenter overlap between thinspiration communities. Values represent the number of commenters in the thinspiration network (N=1086) who posted in the 5 communities (represented by the 5 ovals). Values in overlapping areas indicate the number of commenters who posted in 2 or more communities. The areas of ovals are unscaled and do not represent the size of communities.

Of the 1086 thinspiration commenters, 67.59% (734) also posted on ancillary subreddits within the Pornography: 1 community, compared with 59.85% (650), 46.13% (501), 20.99% (228), and 10.87% (118) in the Pornography: young/small, Pornography: 2, *proED,* and *ProEDmemes* communities, respectively. Figure 5 presents the commenter overlaps between the 5 thinspiration communities.

As shown in Figure 5, a clear distinction was observed between the pornography communities (ie, Pornography: 1, Pornography: 2, and Pornography: young/small) and proeating disorder communities (ie, *ProEDmemes* and *proED*). As such, the overlap between these 2 groups of communities was calculated. Of the 1086 commenters in the thinspiration commenter network, 78.18% (849/1086) only posted on ancillary subreddits within the pornography communities, whereas 16.67% (181/1086) only commented on subreddits within the proeating disorder communities. However, 5.16% (56/1086) of the commenters posted on subreddits within both groups of communities, indicating a small commenter overlap between these groups.

## Discussion

### Summary

Using the example of EDR subreddits, this study demonstrated a methodology that addressed 2 objectives: (1) determine subtypes of forums related to a similar mental health issue and (2) elucidate the characteristics (ie, shared interests) of the subtypes’ commenters by identifying other forums to which they contribute (ie, ancillary subreddits) and investigating the commenter overlap between these subreddits. These 2 objectives were achieved using mixed-methods approaches, comprising techniques that included a network analysis with community detection, text mining, and a manual review of the forums’ topics. Following the identification of 6 subtypes of EDR subreddits, the report focused on 2 specific subtypes—proeating disorder and thinspiration. The proeating disorder commenters also contributed to subreddits relating to the body and eating, mental health, and women, appearance, and mixed topics. Regarding the thinspiration subtype, 78% (849/1086) of the commenters also contributed to pornographic subreddits, whereas 17% (181/1086) also commented on proeating disorder subreddits.

### Principal Findings

#### Objective 1: Determining Subtypes of Eating Disorder–Related Subreddits

Concerning the first objective, through the use of network analyses with community detection and a previously detailed text-mining technique [14], 2 communities of EDR subreddits were identified that differed in terms of their degree of recovery focus (ie, low recovery focus and high recovery focus). The detection of these 2 communities and the relatively small (5%) commenter overlap between them is in line with similar findings relating to the proeating disorder and prorecovery communities on *Twitter* [13]. Furthermore, previous analyses of online data from an EDR online forum indicated that commenters in the least recovery-focused eating disorder stages of change (ie, precontemplation and relapse) used recovery words less frequently than commenters in more recovery-focused stages of change [15]. The findings in this study offer support for this, as recovery was indeed mentioned less frequently in ostensibly proeating disorder subreddits (ie, *MyProAna*, *proED*, *ProEDmemes*, and *thinspo*) than more recovery-focused subreddits (ie, *BingeEatingDisorder*, *bulimia*, *eating_disorders*, *EatingDisorders*, and *fuckeatingdisorders*). Although the text-mining approach used textual data to assess the frequency of words’ occurrence in comment threads, the network analyses with community detection utilized behavioral data (ie, data about the subreddits to which commenters contributed). As the results of the text-mining approach (ie, degree of recovery focus) clearly align with the detected communities (ie, the communities appear to differ on the basis of recovery focus), a strength of this mixed-methods approach is that the 2 distinct techniques appear to provide a degree of convergent validity to each other.

#### Objective 2: Profiling Eating Disorder–Related Commenters Based on Contributions to Ancillary Subreddits

With regard to the second objective, the topics in which commenters on proeating disorder subreddits were interested are in line with other research [10,11,16]. Specifically, commenters on proeating disorder subreddits were also found, unsurprisingly, to be interested in the body, eating, mental health, and appearance. As several identified subreddits were specific to women (eg, *femalefashionadvice* and *TheGirlSurvivalGuide*), the results also supported previous findings that suggest women are more likely to engage with pro-EDR online content [4,9,30].

In contrast to the proeating disorder results, the findings concerning thinspiration commenters were of great surprise. Namely, a clear majority of thinspiration commenters (78%) had also contributed to pornographic subreddits. Furthermore, a specific group of commenters contributed to pornographic subreddits that had names suggesting that women were young (including terms such as *legal* or *18*) or small (including terms such as *tiny*, *petite*, and *small*). This finding is in line with previous research, which concluded that thinspiration images were typically sexually suggestive [30-32]. In fact, one study [32] actually identified pornographic images in their search for thinspiration (and fitspiration) content, although these were excluded from the subsequent analyses. As indicated in this study, the distinction between thinspiration and pornographic material is not clear. As such, it is important that researchers do not exclude specific material (eg, pornographic images) from future analyses as this might lead to a sanitized understanding of thinspiration content. This study therefore highlights an issue of potentially great concern. Specifically, as thinspiration content typically comprises photos of extremely thin women [30], the people submitting this content might not be fully aware of how their content is subsequently used. Speculatively, this lack of a complete understanding might lead to people unintentionally entering into vulnerable situations and would therefore clearly warrant further research.

Although the findings of this study might suggest that people engage with thinspiration for pornographic reasons, it is also possible that engagement with pornography and eating disorder symptomatology are related. For example, pornographic content might be viewed for the purpose of body comparison. However, only 2 studies appear to have explicitly investigated the relationship between pornography use and eating disorder symptomatology, both of which recruited male samples exclusively [33,34]. Given the apparent lack of research investigating this relationship in a female sample and owing to proeating disorder commenters typically being women [9], this topic also represents an important avenue for future research.

Overall, a strength of the findings in this study is that they complement previous studies. Although there are limitations (detailed below) to the current mixed-methods approach, its techniques can compensate for the methodological limitations of the previous studies. For example, survey-based studies concerning users of online forums are unlikely to have representative samples. As the current approach used data concerning all the commenters on public forums, it is not subject to this limitation. As a result, by consolidating the findings generated from these distinct methodological approaches, there can be greater confidence that results do not simply represent an artefact of one particular technique [35].

### Limitations

The findings in this study must be considered in relation to the limitations of the data and methodology. First, it is important to note that the *Reddit* data are unsolicited. Although this represents a strength of the data (eg, the data are not liable to demand characteristics), this also results in a significant amount of noise in the data. Steps were taken to reduce this noise, such as only including 50 ancillary subreddits with a large number and proportion of EDR commenters in the network analyses. This approach identified the most representative subreddits by excluding very small subreddits (many of which had a high proportion but small number of EDR commenters) and very large subreddits (many of which had a large number but low proportion of EDR commenters). However, the effects of other sources of noise in the data are more difficult to mitigate. For example, the same person might have more than one *Reddit* username, which might lead to an underestimation of commenter overlap. Additionally, *bots* (ie, automated software) exist that comment widely on subreddits and which might contribute to an overestimation of commenter overlap. Although strategies exist to identify bots [16,36], these might also exclude actual users. For this reason, bots (except *AutoModerator*, a generic *Reddit* bot) were not excluded, to adopt a conservative approach to our analyses. To minimize the effects of these sources of noise, the conclusions are based on communities of subreddits, rather than individual subreddits. Although not necessarily a limitation of this study, caution should be exercised in generalizing these findings to people who have read, but not commented on, the online content. Exploring communities based on the content that users read would be important but ethically problematic, as this would likely require access to data that are not publicly available. Despite not being able to generalize to the readers of forums, Aardoom et al [37] found that most survey respondents (87.2%) recruited from a prorecovery EDR forum posted content, whereas the remainder only read content. Furthermore, algorithms exist that recommend subreddits in which users might be interested [38]. Therefore, by focusing on commenters, users can be ensured to be actively engaged with the content, rather than being passively exposed to it. Similarly, private subreddits were not included in this study for primarily ethical reasons. However, a private recovery-focused EDR subreddit is advertised on a number of the public EDR subreddits. As such, the results regarding the high recovery-focus subreddits (detailed in Multimedia Appendix 2) might have differed if the private subreddit’s commenters were included. However, the private recovery-focused subreddit is less likely to influence the low recovery-focus analyses presented above.

### Implications

With regard to the approach used for the first objective, the findings in this study have implications for how mental health–related online communities should be conceptualized and investigated in future research. Specifically, when comparing multiple communities, the degree of user overlap between these should be acknowledged. For example, a previous study compared the frequency of fitness tracker mentions between 3 EDR subreddits (ie, *proED, fuckeatingdisorders, and EatingDisorders*) [14]. By considering how these forums overlap, a clearer and more detailed interpretation of the characteristics of these forums’ commenters could be achieved.

Concerning the second objective’s methodological approach, the findings have implications for future research and the design of Web-based psychological interventions for mental health issues. With regard to future research, the approach presented here is entirely reproducible and can be used to explore similar questions in other groups of commenters of particular theoretical interest (eg, relating to other mental health conditions). The methodology could also be easily extended to explore longitudinal—and, therefore, causal—patterns of commenting. The approach is also useful for hypothesis generation and identifying new avenues of research. As detailed above, this study generated a surprising finding, in that over 3 quarters of thinspiration commenters also commented on pornography. As this relationship had not been identified before, it is a clear indicator of how this approach can be used to identify areas that require greater research attention. Concerning implications for psychological interventions, the current approach can identify other topics that are of interest to people commenting on mental health–related online discussion forums. For example, proeating disorder commenters were observed to be also interested in topics such as body, eating, mental health, and appearance. Consequently, these topics confirm the importance of existing EDR intervention focuses (eg, eating and body shape and weight concerns) [39]. These findings could also be used to more accurately tailor interventions to the target population’s characteristics (eg, topics of interest), potentially increasing adherence to the programs [3]. Another implication for psychological interventions is that this approach can identify other forums in which there is a high activity of mental health–related forums’ commenters. In the case of proeating disorder commenters, they were also observed to be active in subreddits including *1200isplenty*, *loseit*, and *progresspics*. As some mental health–related communities (eg, proeating disorder) might be unlikely to promote psychological interventions, the current approach could be utilized to identify the communities in which these users also tend to post. As a result, these communities could be approached to provide an alternative way in which to reach these people and to target prevention-focused interventions.

### Conclusions

In summary, this study has presented a reproducible and primarily data-driven methodology that can be used to (1) identify subtypes of mental health–related forums and (2) identify the interests of the commenters who post on the forums comprising these subtypes. This offers a powerful technique for hypothesis-generation and informing strategies for psychological intervention. Employing different methodologies to explore the same research question is vital to ensure that findings are not solely a result of a particular methodological design [35]. This approach therefore offers one way in which to triangulate methodologically the findings obtained through, for example, previous survey-based research and consequently contributes to a more robust evidence base.

## Appendix

Multimedia Appendix 1 Runnable code.

Multimedia Appendix 2 Supplementary material.

## Abbreviations

EDR: eating disorder–related
