# Supplementary material for: Profiling Commenters on Mental Health–Related Online Forums: A Methodological Example Focusing on Eating Disorder–Related Commenters
Source: JMIR Ment Health. 2019 Apr 22;6(4):e12555. doi: 10.2196/12555 (PMC6658234; doi:10.2196/12555)
Supplement: Multimedia Appendix 2 [file mental_v6i4e12555_app2.pdf]

Supplementary tables

Supplementary table 1. Search terms for eating disorder-related subreddits

**Eating disorder (general)**	**Specific eating disorder diagnostic categories**	**Eating disorder-related online content**
"eating disorder", "eating disorders", "eating disordered", "eatingdisorder", "eatingdisorders", "eatingdisordered", "disordered eating", "disorderedeating", "ed", "eds", "proed", "proeds", "pro-ed", "pro-eds"	"anorexia", "anorexic", "anorexics", "proanorexia", "pro-anorexia", "ana", "proana", "pro-ana", "bulimia", "bulimic", "bulimics", "probulimia", "pro-bulimia", "mia", "promia", "pro-mia", "binge eating", "pica", "rumination disorder", "ednos", "food intake disorder", "arfid", "osfed", "ufed"	"thinspiration", "thinspo", "bonespiration", "bonespo"

Supplementary table 2. Identified eating disorder-related subreddits

EatingDisorders, BingeEatingDisorder, eating_disorders, selectiveeating, EatingDisorderHope, edsupport, fuckeatingdisorders, ProEDmemes, proED, proEDadults, MyProAna, bulimia, ARFID, disorderedeating, eatingdisordered, eatingdisorderstories, EncourageEating, BulimiaRecovery, edpics, antiana, EDFood, thinspocommunity, ProAnaMia, proanaweightloss, malethinspo, EDRecovery_public, TrueThinspo, thinspo, truespo, Thinsporation, proThinspo, AnorexiaNervosa, ProAnorexia, EastBayEDs, SFBayEDs, AustinEDs, Ednos, anorexic, nothaes, ProAnaMPA, ShittyProAna, ProMia, StopPurging, ProBulimia, bingeeating, overcomebingeeating, nobingeing, BingeSupport, Bingeeatsupport, NoBinge

Supplementary analyses

High recovery-focus: Pro-recovery eating disorder

In total, 917 ancillary subreddits had been contributed to by at least 1% (20) of the 1986 commenters associated with the 'pro-recovery eating disorder subreddits' subtype (i.e., *EatingDisorders*, *eating_disorders* and *fuckeatingdisorders*). 50 ancillary subreddits were identified on which 71.20% (1414/1986) of the pro-recovery eating disorder subtype's commenters also posted. The network analysis with community detection is presented in Supplementary figure 1, with a summary of the 50 ancillary subreddits presented in Supplementary table 3.

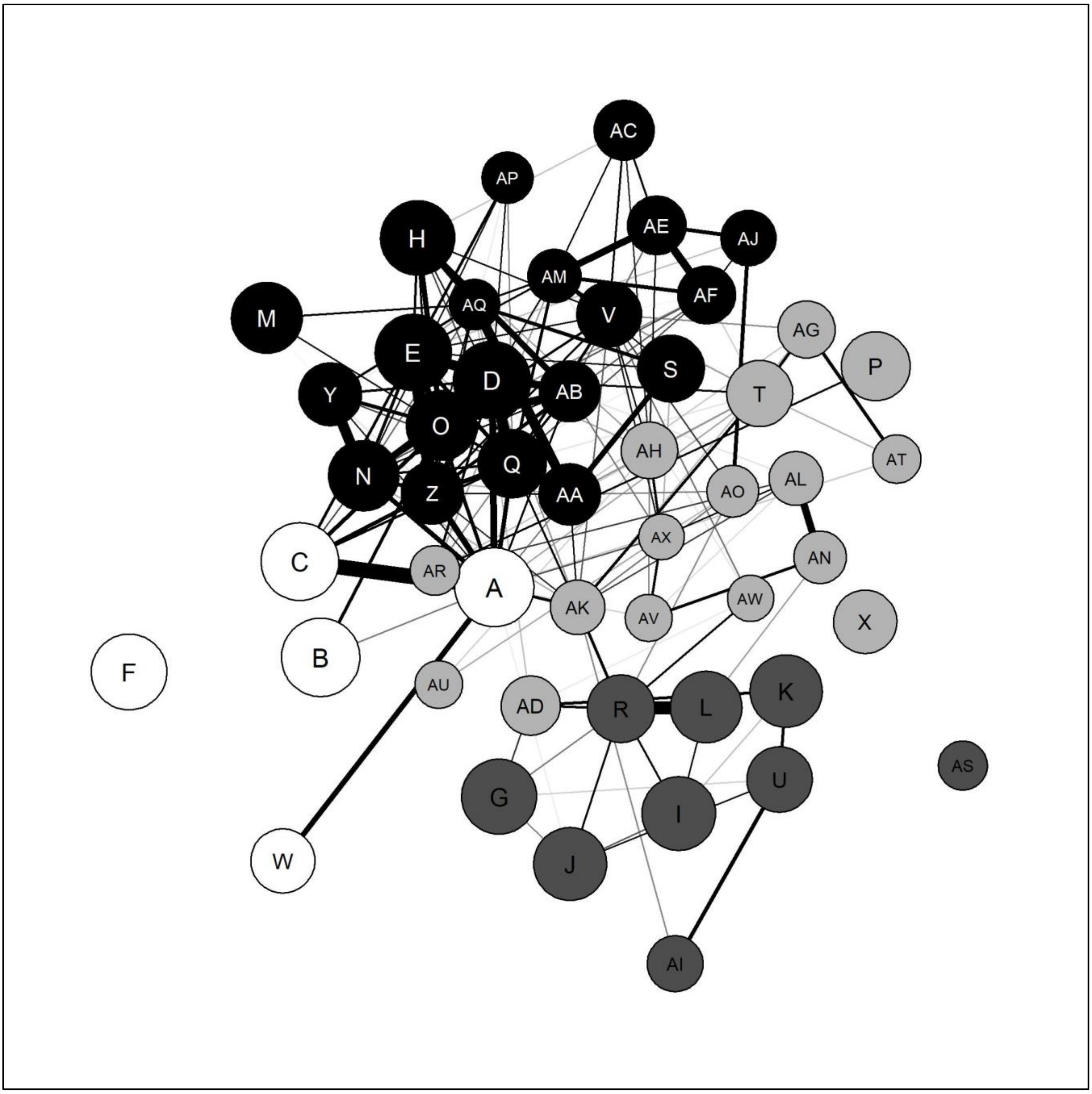

Supplementary figure 1. Pro-recovery eating disorder network. Letters correspond to ancillary subreddits (Supplementary table 3 contains the names and descriptions of the subreddit to which each letter corresponds). Black circles represent community 1 (“Eating/Body/Exercise/Appearance”), dark-gray circles represent community 2 (“Mental health”), light-gray circles represent community 3 (“Mixed”), and white circles represent community 4 (“Eating disorders”). The size of circles represents ancillary subreddit mean rank (larger circle = higher rank), and thickness of the lines represents the mean degree of commenter-overlap between each pair of subreddits (thicker line = larger overlap). No lines representing <0.25 mean commenter-overlap are displayed.

Supplementary table 3. Names and descriptions of ancillary subreddits on which pro-recovery eating disorder commenters posted

Community	Rank	Label	Subreddit name	Description
1[a]	4	D	fatlogic	Weight-loss
1	5	E	1200isplenty	1200kcal daily energy intake
1	8	H	xxfitness	Female fitness
1	13	M	nutrition	Nutrition
1	14	N	fasting	Fasting
1	15	O	loseit	Weight-loss
1	17	Q	fatpeoplestories	Stories about "fat people"
1	19	S	vegetarian	Vegetarianism
1	22	V	TheGirlSurvivalGuide	"A survival guide of "life pro-tips" for the everyday girl"
1	25	Y	intermittentfasting	Fasting
1	26	Z	1200isjerky	1200kcal daily energy intake
1	27	AA	vegan	Veganism
1	28	AB	progresspics	Photos of "body transformations"
1	29	AC	femalefashionadvice	Advice on female fashion
1	31	AE	MakeupAddiction	Make-up addiction
1	32	AF	muacirclejerk	Make-up addiction
1	36	AJ	FancyFollicles	Hair
1	39	AM	SkincareAddiction	"Everything skincare"
1	42	AP	EatCheapAndHealthy	"Eating healthy on a cheap budget"
1	43	AQ	veganfitness	"The place to come and discuss vegan fitness, bodybuilding, and health."
2[b]	7	G	mentalhealth	Mental health
2	9	I	BPD	Borderline personality disorder
2	10	J	selfharm	Self-harm
2	11	K	Anxiety	Anxiety
2	12	L	bipolar	Bipolar disorder
2	18	R	BipolarReddit	Bipolar disorder
2	21	U	depression	Depression
2	35	AI	SuicideWatch	Suicide
2	45	AS	offmychest	"Mutually supportive community where deeply emotional things […] can be told"
3[c]	16	P	raisedbynarcissists	"Support group for people raised by (or being raised by) a narcissistic parent"
3	20	T	badwomensanatomy	Women's anatomy
3	24	X	ptsd	Post-traumatic stress disorder
3	30	AD	OCD	Obsessive compulsive disorder
3	33	AG	actuallesbians	Cis- or trans-lesbians
3	34	AH	AskWomen	Ask women about any subject
3	37	AK	SanctionedSuicide	Suicide
3	38	AL	TrueOffMyChest	Confessions
3	40	AN	confessions	Confessions
3	41	AO	piercing	Piercing
3	44	AR	childfree	People who do not have or want children
3	46	AT	bisexual	Bisexuality
3	47	AU	birthcontrol	"A place to discuss birth control methods."
3	48	AV	AskDocs	Ask doctors about medical subjects

3	49	AW	ADHD	Attention deficit hyperactivity disorder
3	50	AX	askwomenadvice	"a subreddit dedicated to asking women for advice."
4[d]	1	A	proED	Eating disorders [11.75% threads mention recovery]
4	2	B	BingeEatingDisorder	Binge eating disorder [19.08% threads mention recovery]
4	3	C	ProEDmemes	Eating disorders [2.42% threads mention recovery]
4	6	F	bulimia	Bulimia nervosa [39.90% threads mention recovery]
4	23	W	MyProAna	Anorexia nervosa [10.26% threads mention recovery]

[a]Community 1 = *Eating/Body/Exercise/Appearance*

[b]Community 2 = *Mental health*

[c]Community 3 = *Mixed*

[d]Community 4 = *Eating disorders*

As shown in Supplementary figure 1, the algorithm detected 4 communities within the pro-recovery eating disorder subreddits network. Community 1 (black circles) was labelled "Eating/Body/Exercise/Appearance" as the ancillary subreddits mainly related to eating (e.g., *1200isplenty*, *nutrition*, *fasting, vegan*), weight loss and body transformations (e.g., *loseit*, *progresspics*, *fatlogic*), exercise (e.g., *xxfitness*, *veganfitness*), or appearance (e.g., *MakeupAddiction*, *FancyFollicles*). Community 2 (dark-gray circles) was labelled "Mental health" as the subreddits related to mental health conditions (e.g., *Anxiety*, *bipolar*, *depression*), or related issues (e.g., *selfharm*, *SuicideWatch*). Community 3 (light-gray circles) was labelled "Mixed" as the subreddits related to a variety of topics (e.g., *AskWomen*, *OCD*, *piercing*). Community 4 (white circles) was labelled "Eating disorders" as the subreddits related to eating disorders in general (i.e., *proED*, *ProEDmemes*), and specific eating disorder diagnoses (i.e., *BingeEatingDisorder*, *bulimia*, *MyProAna*).

Of the 1414 pro-recovery eating disorder commenters, 58.63% (n=829) also posted on ancillary subreddits within the Eating/Body/Exercise/Appearance community, compared to 50.28% (n=711), 46.18% (n=653) and 36.42% (n=515) in the Mixed, Mental health and Eating disorders communities, respectively. Supplementary figure 2 presents the commenter-overlaps between the four pro-recovery eating disorder communities.

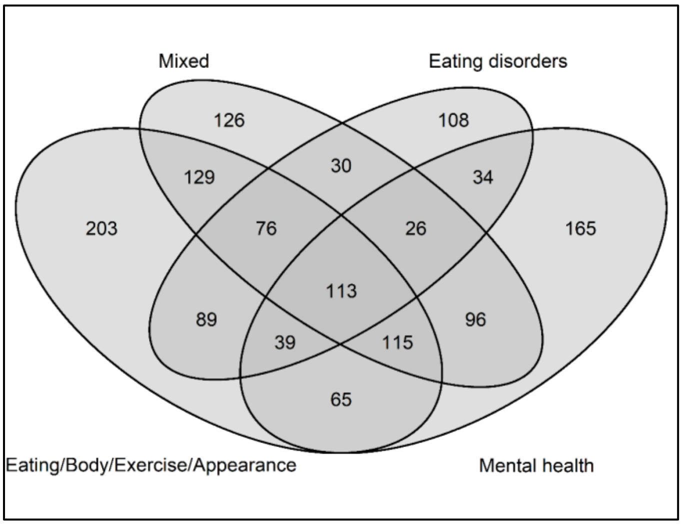

Supplementary figure 2. Commenter-overlap between pro-recovery eating disorder communities. Values represent the number of commenters in the pro-recovery eating disorder network (n=1414) who posted in the 4 communities (represented by the 4 ovals). Values in overlapping areas indicate the number of commenters who posted in 2 or more communities. The areas of ovals are unscaled and do not represent the size of communities.

High recovery-focus: Pro-recovery binge eating disorder

In total, 1830 ancillary subreddits had been contributed to by at least 1% (25) of the 2520 commenters associated with the ‘pro-recovery binge eating disorder’ subtype (i.e., *BingeEatingDisorder*). 50 ancillary subreddits were identified on which 70.44% (1775/2520) of the pro-recovery binge eating disorder subtype’s commenters also posted. The network analysis with community detection is presented in Supplementary figure 3, with a summary of the 50 ancillary subreddits presented in Supplementary table 4.

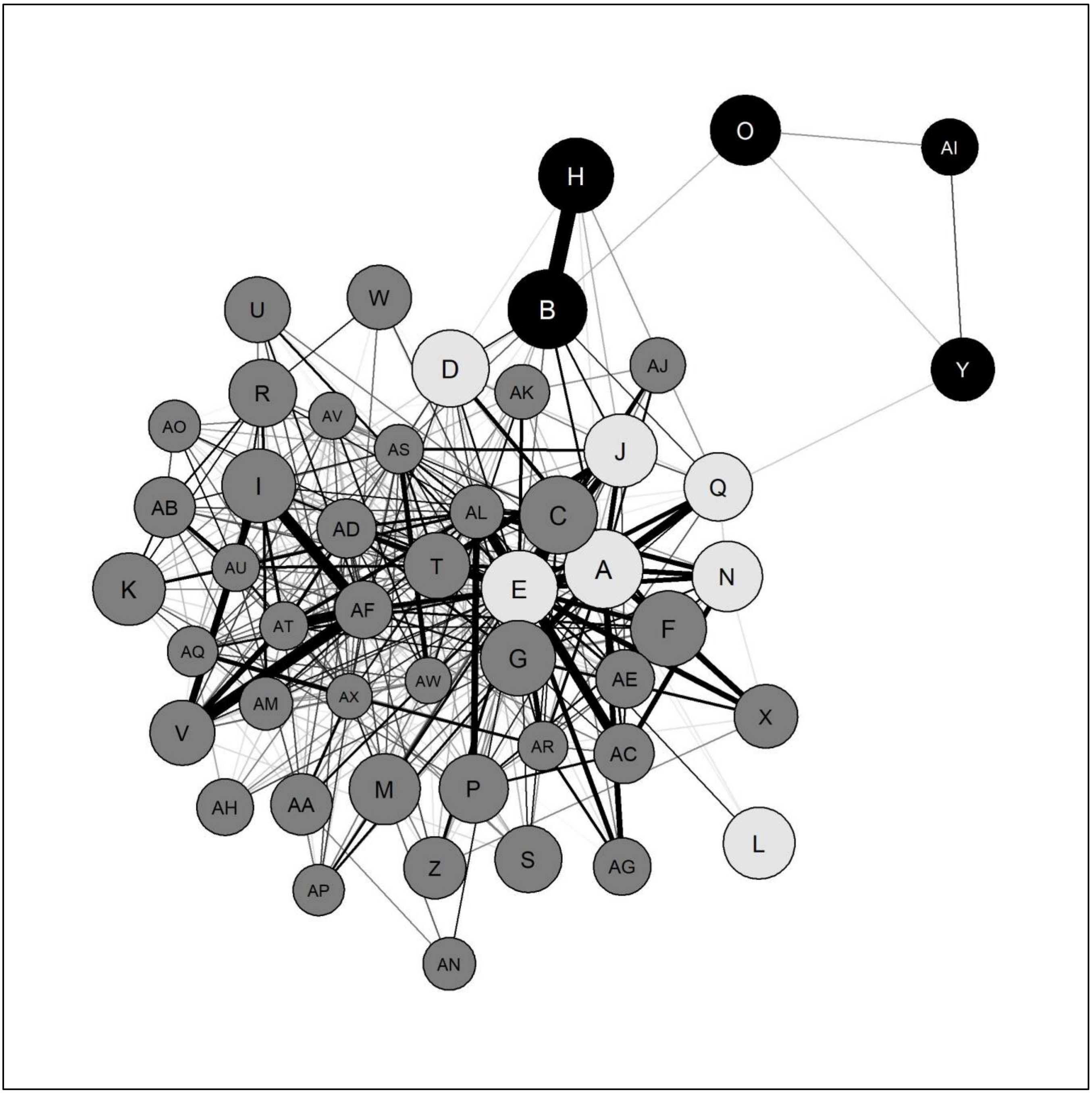

Supplementary figure 3. Pro-recovery binge eating disorder network. Letters correspond to ancillary subreddits (Supplementary table 4 contains the names and descriptions of the subreddit to which each letter corresponds). Black circles represent community 1 (“Eating disorders”), dark-gray circles represent community 2 (“Mixed”), and light-gray circles represent community 3 (“Eating/Body”). The size of circles represents ancillary subreddit mean rank (larger circle = higher rank), and thickness of the lines represents the mean degree of commenter-overlap between each pair of subreddits (thicker line = larger overlap). No lines representing <0.25 mean commenter-overlap are displayed.

Supplementary table 4. Names and descriptions of ancillary subreddits on which pro-recovery binge eating disorder commenters posted

Community	Rank	Label	Subreddit name	Description
1[a]	2	B	proED	Eating disorders [11.75% threads mention recovery]
1	8	H	ProEDmemes	Eating disorders [2.42% threads mention recovery]
1	15	O	fuckeatingdisorders	Eating disorders [46.59% threads mention recovery]
1	25	Y	eating_disorders	Eating disorders [32.78% threads mention recovery]
1	35	AI	EatingDisorders	Eating disorders [46.72% threads mention recovery]
2[b]	3	C	xxfitness	Female fitness
2	6	F	intermittentfasting	Fasting
2	7	G	progresspics	Photos of "body transformations"
2	9	I	xxketo	Female keto diet
2	11	K	BPD	Borderline personality disorder
2	13	M	TheGirlSurvivalGuide	"A survival guide of "life pro-tips" for the everyday girl"
2	16	P	PlantBasedDiet	Plant based diet
2	18	R	mentalhealth	Mental health
2	19	S	Paleo	Paleo diet
2	20	T	fatpeoplestories	Stories about "fat people"
2	21	U	PCOS	Polycystic Ovarian Syndrome
2	22	V	ketorecipes	Keto recipes
2	23	W	Anxiety	Anxiety
2	24	X	1500isplenty	1500kcal daily energy intake
2	26	Z	EOOD	“Exercise Out Of Depression”
2	27	AA	getdisciplined	“A subreddit for people who have issues with procrastination, motivation, and discipline.”
2	28	AB	bipolar	Bipolar disorder
2	29	AC	veganrecipes	Vegan recipes
2	30	AD	C25K	Couch to 5k running programme
2	31	AE	vegetarian	Vegetarianism
2	32	AF	keto	Keto diet
2	33	AG	EatCheapAndHealthy	"Eating healthy on a cheap budget"
2	34	AH	Hair	Hair
2	36	AJ	femalefashionadvice	Advice on female fashion
2	37	AK	FancyFollicles	Hair
2	38	AL	veganfitness	“The place to come and discuss vegan fitness, bodybuilding, and health.”
2	39	AM	OCD	Obsessive compulsive disorder
2	40	AN	DecidingToBeBetter	“A force for self-improvement, goodness, and togetherness that helps humanity eliminate evil.”
2	41	AO	ADHD	Attention deficit hyperactivity disorder
2	42	AP	yoga	Yoga
2	43	AQ	curlyhair	Hair
2	44	AR	MealPrepSunday	Meal preparation
2	45	AS	muacirclejerk	Make-up addiction
2	46	AT	ketogains	“ A community driven exploration into the pursuit of physical excellence via ketosis.”
2	47	AU	CPTSD	Complex post traumatic stress disorder
2	48	AV	bulletjournal	Method of organisation
2	49	AW	RedditLaqueristas	Nail polish

2	50	AX	internetparents	"Ask the internet about things your parents never taught you."
3[c]	1	A	1200isplenty	1200kcal daily energy intake
3	4	D	fasting	Fasting
3	5	E	loseit	Weight-loss
3	10	J	fatlogic	Weight-loss
3	12	L	nutrition	Nutrition
3	14	N	vegan1200isplenty	1200kcal daily energy intake (vegan)
3	17	Q	1200isjerky	1200kcal daily energy intake

[a]Community 1 = *Eating disorders*

[b]Community 2 = Mixed

[c]Community 3 = *Eating/Body*

As shown in Supplementary figure 3, the algorithm detected three communities within the pro-recovery binge eating disorder network. Community 1 (black circles) was labelled "Eating disorders" as the ancillary subreddits related to eating disorders in general (e.g., *proED*, *EatingDisorders*). Community 2 (dark-gray circles) was labelled "Mixed" as it comprised a variety of subreddits (e.g., *xxfitness*, *mentalhealth*, *ketorecipes*). Community 3 (light-gray circles) was labelled "Eating/Body" as the subreddits mainly related to restrictive eating (e.g., *1200isplenty*, *fasting*), or weight loss (e.g., *loseit*, *fatlogic*).

Of the 1775 pro-recovery binge eating disorder commenters, 80.11% (*n*=1422) also posted on ancillary subreddits within the Mixed community, compared to 64.56% (*n*=1146) and 23.55% (*n*=418) in the Eating/Body and Eating disorders communities, respectively. Supplementary figure 4 presents the commenter-overlaps between the three pro-recovery binge eating disorder communities.

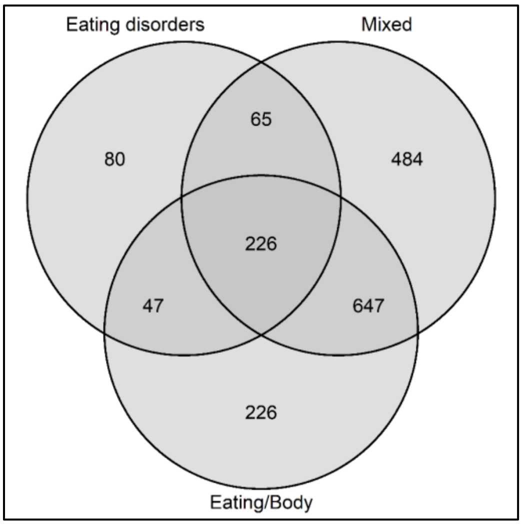

Supplementary figure 4. Commenter-overlap between pro-recovery binge eating disorder communities. Values represent the number of commenters in the pro-recovery binge eating disorder network (n=1775) who posted in the 3 communities (represented by the 3 circles). Values in overlapping areas indicate the number of commenters who posted in 2 or 3 communities. The areas of circles are unscaled and do not represent the size of communities.
